# Agentic and LLM-Based Multimodal Anomaly Detection: Architectures, Challenges, and Prospects

**DOI:** 10.3390/s26082330

**Published:** 2026-04-09

**Authors:** Mohammed Ayalew Belay, Amirshayan Haghipour, Adil Rasheed, Pierluigi Salvo Rossi

**Affiliations:** 1 Department of Electronic Systems, Norwegian University of Science and Technology, 7034 Trondheim, Norway; 2 Department of Engineering Cybernetics, Norwegian University of Science and Technology, 7034 Trondheim, Norway

**Keywords:** agentic anomaly detection, agents, large language models, multimodal, cross-modal fusion, agentic AI

## Abstract

Anomaly detection is crucial in maintaining the safety, reliability, and optimal performance of complex systems across diverse domains, such as industrial manufacturing, cybersecurity, and autonomous systems. While conventional methods typically handle single data modalities, recently, there has been an increase in the application of multimodal detection in dynamic real-world environments. This paper presents a comprehensive review of recent research at the intersection of agentic artificial intelligence and large language-based multimodal anomaly detection. We systematically analyze and categorize existing studies based on the agent architecture, reasoning capabilities, tool integration, and modality scope. The main contribution of this work is a novel taxonomy that unifies agentic and multimodal anomaly detection methods, alongside benchmark datasets, evaluation methods, key challenges, and mitigation strategies. Furthermore, we identify major open issues, including data alignment, scalability, reliability, explainability, and evaluation standardization. Finally, we outline future research directions, with a particular emphasis on trustworthy autonomous agents, efficient multimodal fusion, human-in-the-loop systems, and real-world deployment in safety-critical applications.

## 1. Introduction

Anomaly detection is becoming increasingly crucial in ensuring the reliability, security, and optimal performance of complex systems across a wide range of domains. From industrial manufacturing [1,2] and financial services [3] to healthcare [4], cybersecurity [5,6,7,8,9], and autonomous systems [10], the early identification of abnormal patterns plays a vital role in preventing costly failures, supporting timely decision-making, and mitigating risks. At its core, anomaly detection involves identifying data patterns that deviate from expected or normal behavior [11,12]. These anomalies often signify critical events or faults; for instance, unusual network traffic may suggest a cyberattack [5,13], aberrant sensor readings could indicate impending equipment failure [14,15], and outlier financial transactions might reveal fraudulent activity [16,17]. Because anomalies typically represent rare but significant occurrences, anomaly detection has been extensively studied in the past few years [18,19,20].

Conventional anomaly detectors have typically modeled a single data modality and a fixed normal profile (e.g., via statistical thresholds, one-class SVMs, nearest neighbors, or density models). However, the rise in data complexity and volume has challenged these methods. Deep learning has become a dominant paradigm in recent years. Pang et al. [21] highlight that deep anomaly detection (using autoencoders, GANs, normalizing flows, etc.) has emerged as a critical direction with unique problem complexities [22]. These methods can learn rich representations of normal data, but most still assume a single input modality or sensor [23,24]. As a result, purely single-modal approaches may miss anomalies that only manifest when multiple data sources are considered together. To address this, multimodal anomaly detection has gained prominence. Modern systems often have heterogeneous sensors (e.g., image, audio, thermal, etc.), and fusing their information can improve robustness [25,26]. Lin et al. [27] present unsupervised industrial anomaly detection that integrates RGB images with 3D scans and yields much better performance than either alone. Similarly, Li et al. [28] introduce MulSen-AD, a multi-sensor anomaly dataset that unifies RGB cameras, laser scans, and infrared thermography. Other domains show analogous trends: in robotics, for example, anomaly detectors now combine proprioceptive signals (forces, motions) with visual language models to capture both mechanical and environmental failures [29]. In surveillance, the fusion of video and audio cues has been explored to capture anomalies that are invisible in vision alone. In general, modern anomaly detection increasingly relies on multimodal inputs to capture complex anomalies that single sensors might miss [28].

Another recent development is agentic AI—autonomous, goal-driven AI agents that can reason, plan, and use tools with minimal human intervention [30]. Agentic AI is designed to pursue complex goals with characteristics such as adaptability and decision-making under uncertainty [31]. Unlike conventional or purely generative AI, agentic systems are built to operate in uncontrolled environments, setting their own subgoals and using external tools or information as needed [32]. Many modern large language model (LLM) applications (e.g., AutoGPT, ReAct agents, and tool-augmented chatbots) fall under this paradigm. Agentic anomaly detection enables such systems to incorporate reasoning, context, and tool use into the detection process. For instance, LLM-based agents can utilize contextual knowledge, parse unstructured data (such as system logs or maintenance notes), and iteratively refine anomaly criteria. Recently, several agentic and multimodal anomaly detection methods have been proposed. Russell-Gilbert et al. [33] demonstrate the framing of time-series anomaly detection as a language task, using a pre-trained LLM to interpret sensor readings in context without retraining. Liu et al. [22] present a method that combines generative diffusion models with LLMs for human-interpretable anomaly explanations and contextual reasoning. In practice, agentic anomaly systems autonomously query multiple data sources (e.g., visual feeds, databases, knowledge graphs) and apply chain-of-thought reasoning to decide if an anomaly is significant.

Several recent works on anomaly detection provide extensive surveys of conventional and deep learning-based methods. Chandola et al. [11] established foundational taxonomies across multiple application domains. Chalapathy et al. [34] and Pang et al. [21] specifically addressed deep learning techniques, categorizing key neural architectures and methodological advances. Domain-specific reviews such as Cook et al. [35] target IoT-based systems, while Erhan et al. [36] focus on sensor-centric anomaly detection. Moreover, Choi et al. [37] and Garg et al. [38] provide practical guidelines, benchmarking methodologies, and comparative evaluations. However, despite this progress, no existing survey comprehensively focuses on the intersection of agentic and multimodal anomaly detection.

In this paper, we fill this gap by providing a comprehensive and up-to-date synthesis of recent advancements in anomaly detection that leverage both agentic AI systems (autonomous, reasoning-capable agents) and multimodal data integration frameworks. We present a unified taxonomy, compare key methods, identify prevailing trends, and outline open research challenges in this rapidly evolving field. Specifically, the contributions of this work are as follows:

- We provide a structured survey of anomaly detection approaches that incorporate both agentic AI and multimodal data fusion.
- We introduce a novel taxonomy to classify existing methods based on the agent architecture (single-agent vs. multi-agent), reasoning capabilities, tool integration, and modality scope.
- We review recent benchmark datasets and evaluation methods for multimodal anomaly detection.
- We present key challenges and summarize mitigation strategies and future directions in agentic and multimodal anomaly detection.

The remainder of the paper is organized as follows. Section 3 and Section 4 review multimodal anomaly detection methods and fusion techniques. Section 2 discusses agentic anomaly detection and classifies agent architectures and capabilities. Section 5 summarizes multimodal anomaly detection datasets and benchmarks. Section 6 highlights key challenges, mitigation strategies, and open research problems. Finally, Section 7 concludes the paper and outlines future research directions.

## 2. Agentic Anomaly Detection

Agentic anomaly detection (AAD) extends conventional anomaly detection paradigms by integrating autonomy, reasoning, and tool use into detection pipelines. These systems, often built upon large language models (LLMs) or reinforcement learning (RL) agents, enable the dynamic decomposition of complex tasks, the contextual interpretation of multimodal signals, and the adaptive orchestration of detection tools and models. In conventional anomaly detection, a scoring function s(x) is defined for input *x* and flags an anomaly if s(x) exceeds a threshold τ (or equivalently if the estimated probability of *x* being generated by a normal data distribution falls below some value α). Unlike static detectors, agentic systems possess the ability to interact with external knowledge sources, communicate between agents, and iteratively refine their predictions based on observations and reasoning outcomes. Agentic systems transform the one-shot detection function into an interactive process that can incorporate external knowledge and multi-step reasoning to improve the accuracy and explainability. We categorize agentic AD methods along three principal paradigms: agent architecture, agent capabilities, and modality scope. Table 1 summarizes representative agentic AD frameworks and their attributes.

### 2.1. Architectures

Agentic anomaly detection frameworks can be broadly categorized into two architectural paradigms: *single-agent systems* and *multi-agent systems*. A single-agent system relies on one central intelligent agent (typically an LLM or a single RL policy), responsible for the end-to-end anomaly detection task. In contrast, a multi-agent system decomposes the detection pipeline into specialized components, each operated by a dedicated agent with clearly defined roles and responsibilities. Below, we discuss these architectures and their internal operation.

#### 2.1.1. Single-Agent Systems

Single-agent AD frameworks operate with a single decision-making entity and often take on one of two roles: (i) *a tool-augmented orchestrator* or (ii) *a reasoner*. In the first setup, an LLM-based agent serves as a central controller that interfaces with external analytics tools, databases, or knowledge graphs to enrich its decision-making. The agent functions as an orchestrator that queries tools and synthesizes their results before making a final anomaly decision. For example, Timms et al. [43] introduce a GPT-4-based maritime anomaly detection system that augments LLM capabilities with access to sensor logs, environmental databases, and a domain-specific knowledge graph. In the second role, the agent utilizes the inferential and few-shot reasoning abilities of LLMs to derive human-interpretable rules or patterns from normal data and then uses these rules to detect anomalies in new data. Yang et al. [44] propose *AnomalyRuler*, a two-stage zero-shot AD framework for video streams using GPT-4V. The agent first induces behavioral rules from a few-shot set of normal videos and then applies these rules to detect and explain anomalies in unseen data. Similarly, Zhang and colleagues [45] introduce *LogSAD*, which combines the Generative Pre-Trained Transformer with Vision (GPT-4V), Contrastive Language–Image Pre-Training (CLIP), and the Segment Anything Model to form a rule-based vision language model (VLM). By generating compositional rules aligned with industrial visual anomalies, LogSAD detects structural defects beyond the reach of conventional pixel-level models without model fine-tuning.

#### 2.1.2. Multi-Agent Systems

Multi-agent systems adopt a distributed approach where multiple specialized agents cooperate to accomplish the anomaly detection task. The agents may operate in sequence or in parallel and can even oversee each other’s performance. We distinguish two common patterns: collaborative pipelines, in which agents collectively execute different stages of a detection workflow, and oversight architectures, in which certain agents monitor or verify the actions of others to ensure reliability and coherence.

- *Collaborative Pipelines*: In a collaborative multi-agent pipeline, each agent is assigned a specific subtask (data preprocessing, feature extraction, anomaly scoring, explanation, etc.), and the output of one agent feeds into the input of the next. If we label the agents $A_{1},A_{2},\ldots ,A_{N}$ as they appear in the pipeline, the overall detection function can be viewed as a composite of their operations:
  (1) $$y=A_{N}\left(A_{N-1}\left(\cdots A_{2}\left(A_{1}\left(x\right)\right)\cdots \right)\right),$$
  where *x* is the initial input and *y* is the final anomaly decision or score. Each $A_{i}$ focuses on a delimited aspect of the task, and their coordination can be managed via an LLM-based planner. For example, Gu et al. [39] propose *ARGOS*, a multi-agent time-series AD framework that autonomously generates, validates, and refines detection rules using collaborative agents. Similarly, Yang et al. [40] introduce *AD-AGENT*, which employs a team of LLM agents to interactively build a complete anomaly detection pipeline from a high-level user instruction. Not all pipelines are strictly sequential; some agents might work in parallel on different data streams or features. For instance, Qin et al. [49] propose *MAS-LSTM*, where multiple LSTM-based detector agents each monitor a different subset of IIoT sensor streams, and anomalies are decided by voting or averaging their scores.
- *Oversight Agents:* As agentic systems become more complex, ensuring reliability and consistency is essential. Oversight architectures introduce dedicated agents that monitor and verify the outputs of task-oriented agents, effectively performing anomaly detection on the multi-agent system itself. These agents catch logical inconsistencies, hallucinations, or coordination failures that could compromise anomaly decisions. Formally, let $\left\{o_{i}\right\}$ be a collection of observations or outputs from various agents during an investigation; the oversight agent computes a consistency score or logical coherence measure $C(o_{1},o_{2},\ldots ,o_{k})$ over these. If *C* falls below a threshold (indicating incoherence or inconsistency), the oversight agent flags a meta-anomaly and can intervene (e.g., by resetting certain agents or requesting additional information). This adds a layer of fault tolerance and accountability to the agentic AD pipeline. For example, He et al. [41] propose *SentinelAgent*, which deploys an LLM oversight agent to supervise a team of collaborating agents. Similarly, in the *Audit-LLM* framework for security logs [42], a critic agent reviews the decisions made by a detector agent and either approves them or asks for refinement, ensuring high-stakes anomaly alerts.

### 2.2. Agent Capabilities

**Figure 1:**
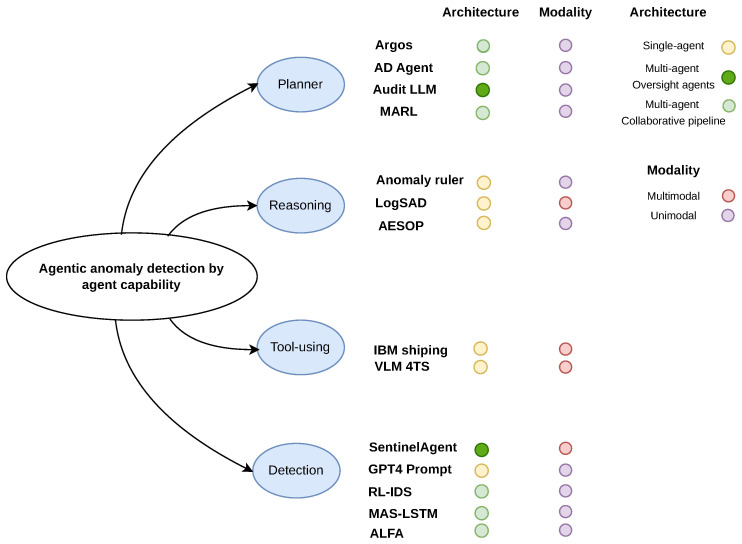
Agentic anomaly detection.

The rapid development of autonomous agents has led to frameworks with varying depths of reasoning and autonomy. Plaat et al. [32] categorize agents by their abilities to reason, act, and interact, emphasizing how agents decompose tasks, use external tools, and collaborate towards goals. Building on this idea, we classify agentic anomaly detection systems by their functional capabilities, resulting in four major categories: *detection-only agents*, *reasoning agents*, *tool-using agents*, and *planner agents*, as shown in Figure 1. Table 2 summarizes these categories, including key examples, strengths, and limitations.

#### 2.2.1. Detection-Only Agents

These agents operate similarly to traditional anomaly detectors, except that an LLM (or other foundation model) is used to directly produce anomaly labels or scores from the raw input, without performing explicit multi-step reasoning or tool use. In other words, the agent’s policy is essentially a single-step mapping f:x↦y. For instance, one can prompt a large language model to output whether an input is anomalous or not in a zero-shot approach:

(2) y=LLM(Prompt(x)),

where the prompt might be a template converting *x* (which could be a time series, log message, etc.) into a descriptive question for the LLM, and *y* is the model prediction. Detection-only agents forego complex reasoning in favor of direct inference, making them straightforward to deploy and fast at runtime. Yang et al. [54] introduce AD-LLM, the first benchmark to evaluate the capabilities of LLMs for zero-shot anomaly detection, data augmentation, and model selection. Cao et al. [55] introduce TAD-Bench, a benchmark that uses state-of-the-art language model embeddings (from BERT, GPT, etc.) combined with classic anomaly detection algorithms (like isolation forests and autoencoders) for text anomalies. In the time-series domain, Alnegheimish et al. [53] propose SigLLM, a framework that serializes time-series data into textual descriptions and then uses an LLM (through prompting) to identify anomalies. Another line of work directly evaluates the zero-shot detection prowess of GPT-4 and similar models on various data. Dong et al. [52] tested GPT-4 on time-series anomaly detection by providing the model with sequences of values as input and asking it to highlight anomalies. Similarly, Derakhshan et al. [56] demonstrate that GPT-4o can effectively detect rework anomalies in business process event logs, achieving high accuracy across different anomaly distributions using zero-shot, one-shot, and few-shot prompting strategies. In general, detection-only agents benefit from the rich prior knowledge of foundation models and their ability to generalize from just a description of the task. They remain useful where low-latency or low-complexity anomaly flagging is required, especially for high-throughput tasks such as real-time sensor screening or log filtering. However, they are prone to the well-known pitfalls of LLMs, such as hallucination and limited transparency.

#### 2.2.2. Reasoning Agents

Reasoning agents incorporate intermediate inference or rule induction into the anomaly detection process, enabling more interpretable and generalizable decisions. Instead of mapping input *x* directly to an output *y*, these agents construct a reasoning chain $c_{1:T}$, which guides their final decision:

(3) $$c_{1:T}=\pi \left(x\right),\;y=f\left(x,\;c_{1:T}\right),$$

where $c_{1:T}$ represents a sequence of intermediate steps (e.g., logical inferences, verbalized rules, or structured descriptions), π is a reasoning policy, and *f* is a decision function that uses both the input and reasoning trace. These steps act as latent variables, often human-readable, providing explainability. For instance, Yang et al. [44] propose *AnomalyRuler*, a zero-shot video anomaly detection framework using GPT-4V. The agent first observes normal video clips to induce temporal and behavioral rules and then applies these rules to new data to detect anomalies via chain-of-thought reasoning. Zhang et al. [45] combine GPT-4V and CLIP/SAM to perform logical and structural reasoning over industrial images without any training. Reasoning agents are particularly valuable in domains where explainability or concept-level anomaly interpretation is critical (e.g., surveillance, medical imaging). However, they heavily rely on well-constructed prompts and representative normal examples. Long reasoning chains may also introduce error propagation.

#### 2.2.3. Tool-Using Agents

Tool-using agents augment their own capabilities by calling external tools (knowledge graphs, search engines, anomaly scoring modules, or third-party APIs) during the anomaly detection process. These agents treat tool outputs as additional observations that inform their final decisions. We can describe a tool-using agent’s operation in two phases: (1) deciding which tool to use and what query to send and (2) integrating the tool’s result into anomaly inference. If $q_{i}$ is a query formulated by the agent and $T_{1},T_{2},\ldots ,T_{k}$ denotes external tool functions, the final decision could be represented as

(4) $$y=A\left(x;T_{1}\left(q_{1}\right),T_{2}\left(q_{2}\right),\ldots ,T_{k}\left(q_{k}\right)\right),$$

where *x* is the input data (or its derived features), *A* is the tool-using agent, and *y* is the output anomaly decision or score. The LLM agent *A* thus combines the raw input with tool outputs $T_{i}\left(q_{i}\right)$ to produce a context-informed anomaly assessment. For example, Timms et al. [43] propose a GPT-4-based maritime anomaly detector that ingests real-time vessel sensor logs with queries from external maritime knowledge graphs and weather APIs. He et al. [41] propose SentinelAgent, a framework that uses a graph-based oversight model where agents query external APIs and maintain long-term tool states. He et al. [46] introduce VLM4TS, which reformulates time series into vision language representations and then uses GPT-4V and retrieval tools to reason about anomalies. Gu et al. [39] propose ARGOS, which integrates LLM-generated rules with classical rule-based methods for time-series anomaly detection in cloud infrastructure. Tool-using agents blend the power of LLMs with domain-grounded computation. They are suitable for hybrid setups such as industrial pipelines with structured anomaly detection.

#### 2.2.4. Planner Agents

Planner agents orchestrate multi-step workflows for anomaly detection, combining reasoning, action selection, and coordination across tools or subagents. These agents are capable of decomposing high-level goals into executable plans, managing task dependencies, and adapting dynamically to intermediate outcomes. Formally, a planner seeks an optimal action sequence $\Pi^{*}$ from a space of possible action sequences S that maximizes some utility (e.g., detection accuracy or information gain):

(5) $$\Pi^{*}\left(x\right)=\arg\max_{\Pi \in S}U\left(\Pi ;\;x\right),$$

where $\Pi =[a_{1},a_{2},\ldots ,a_{n}]$ is a sequence of actions, such as invoking tools, querying subagents, or acquiring new data. In practice, planners often use heuristic or learned strategies rather than solving this optimization exactly, but the formulation emphasizes the agent’s need to anticipate consequences and coordinate across steps. A representative example is *Audit-LLM* [42], a framework for insider threat detection in audit logs. An LLM-based planner (coordinator) oversees three roles: a *decomposer* breaks down high-level questions into queries, an *executor* runs them on the log data, and a *critic* validates the results. The planner governs when to trigger deeper analysis or terminate the investigation, enabling adaptive depth. In the financial domain, Park et al. [3] design a multi-agent planner that coordinates tasks like searching external sources (e.g., news or market data), running anomaly checks, and synthesizing findings into a final report. *ARGOS* [39] also includes a planning component that iteratively refines anomaly detection rules by designing and validating experiments. This allows the system to not only detect but also improve its detection logic over time. Planner agents are best suited for real-world, multi-stage, and high-stakes AD scenarios where the detection task requires exploration, hypothesis testing, or intervention. However, their complexity presents challenges: debugging multi-step plans is difficult, coordination errors may arise, and latency increases with plan depth. Moreover, ensuring safe and reliable behavior in these agents remains an open problem.

**Figure 2:**
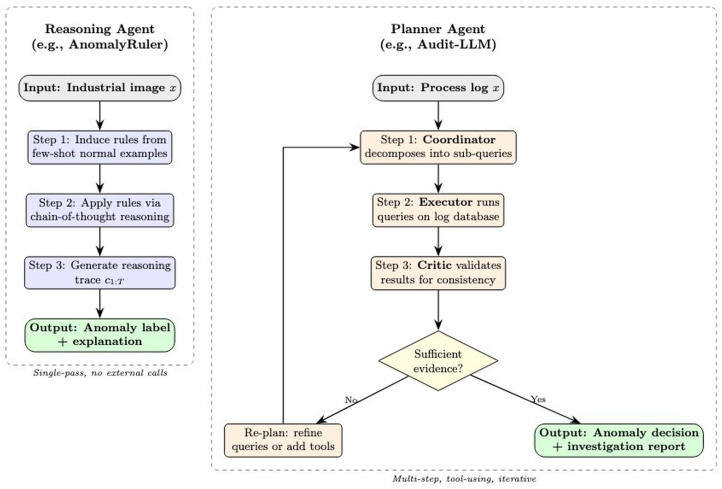
Reasoner vs. planner agent comparison.

Although reasoning agents and planner agents both go beyond simple detection by incorporating intermediate inference steps, they differ fundamentally in *scope*, *control flow*, and *adaptivity*. A reasoning agent performs a single-pass inference chain: given an input, it derives a sequence of logical steps (e.g., rule induction or chain-of-thought) and produces a final anomaly decision without revisiting earlier steps or invoking external actions. In contrast, a planner agent orchestrates a multi-step *workflow* that may include branching, looping, tool invocation, delegation to subagents, and dynamic replanning based on intermediate outcomes. Table 3 summarizes the key distinctions, and Figure 2 illustrates the contrasting processing pipelines through a concrete industrial anomaly detection scenario.

### 2.3. Modality Integration

Modern anomaly detection (AD) systems are increasingly required to handle diverse data sources from heterogeneous sensor streams or data sources. Agentic AD methods can therefore be distinguished by their modality scope. A unimodal agent specializes in a single data modality, whereas a multimodal agent is designed to fuse and reason over multiple modalities.

#### 2.3.1. Unimodal Agentic Detectors

Unimodal agents specialize in one modality, such as vision, time series, or logs. These agents are particularly effective in domains where anomalies are confined to a single data type. For example, Dong et al. [52] treat GPT-4 as a time-series agent by prompting it with numeric sequences to identify abnormal patterns. Zhu et al. [48] introduce ALFA, a vision language model that performs zero-shot image anomaly detection by generating visual descriptions from image inputs and identifying deviations, effectively acting as a vision-centric unimodal agent. Audit-LLM [42], focused on event logs and metadata, operates entirely within the textual domain, making it a log/text unimodal agent. Unimodal agents provide simpler architectures, lower data alignment complexity, and efficient computation due to reduced input dimensionality. However, they are less effective when anomalies emerge from cross-modal interactions.

#### 2.3.2. Multimodal Agentic Detectors

Multimodal agents process and reason over multiple data modalities, such as vision, time series, text, or structured metadata, to detect anomalies that are only apparent through their interaction. These agents must solve two core challenges: (i) fusing features from heterogeneous modalities and (ii) performing cross-modal reasoning to detect inconsistencies or correlations. These agents must align features across modalities and perform joint reasoning. A common solution is to extract features from each modality and combine them in a shared representation:

(6) $$z^{\left(i\right)}=\phi_{i}\left(x^{\left(i\right)}\right),\;z=\left[z^{\left(1\right)},z^{\left(2\right)},\ldots ,z^{\left(M\right)}\right],$$

where $\phi_{i}$ is a modality-specific encoder and [·] denotes fusion (e.g., concatenation or attention). An anomaly score s(z) or decision function is then applied, often via an LLM or neural classifier. For example, *SentinelAgent* [41] fuses textual inter-agent messages with structured environment states to detect coordination failures. *LogSAD* [45] jointly analyzes industrial images and machine logs to detect visual–textual inconsistencies. *VLM4TS* [46] reformulates time series as image–text pairs and analyzes them using GPT-4V, effectively treating time-series anomalies as vision language problems. Similarly, the maritime anomaly detection system by Timms et al. [43] combines numeric sensor data with knowledge graph context to reason about situational normality. Multimodal agents are particularly useful in ambiguous or noisy settings, where cross-modal evidence reduces uncertainty. Mathematically, such agents implicitly learn the joint distribution $P(x^{\left(1\right)},x^{\left(2\right)},\ldots ,x^{\left(M\right)})$, enabling them to detect anomalies in the co-occurrence structure. These agents also enhance explainability by pointing to complementary evidence. However, they come with added complexity: synchronization across modalities, higher model capacity, and potential complexity if one modality introduces noise.

## 3. Multimodal Anomaly Detection

Multimodal anomaly detection (MAD) methods detect anomalous patterns by jointly analyzing data from heterogeneous or multiple modalities, such as images, video, audio, text/logs, and sensor streams [27,57]. Traditional anomaly detection methods often operate within a single modality, but recent advances in deep learning and foundation models have enabled the fusion of heterogeneous sources, leveraging cross-modal context to improve robustness, interpretability, and generalization [58,59]. The emergence of large multimodal models (LMMs), vision language models (VLMs), and LLMs with reasoning capabilities has further accelerated this shift, enabling more powerful and generalizable anomaly detection pipelines. Recently, multimodal AD methods have been utilized in industrial inspection, video surveillance, autonomous systems, and more. In industrial quality control, multimodal AD often fuses visual (RGB camera) and 3D information (depth or point clouds) to detect manufacturing defects. Depth data can reveal surface anomalies or misalignments invisible in color images, significantly improving detection under varying lighting or textures [60]. In multimodal video AD systems, video frames and synchronized audio are combined so that visual cues capture physical actions, while audio can signal alarms [61]. In robotics and autonomous systems, heterogeneous modalities are fused to detect anomalies in robot behavior or environment interactions [29]. We categorize recent advances in MAD into three primary paradigms: foundation models, cross-modal fusion models, and multimodal augmentation and synthesis. Table 4 outlines representative recent methods, their modalities, and key innovations.

### 3.1. Foundation Models

Foundation models such as CLIP, GPT-4V, and MiniGPT-4 are increasingly being applied to MAD as unified encoders and interpretable reasoning agents. These models embed multimodal inputs into shared semantic spaces, enabling zero-shot or few-shot anomaly detection with natural language explanations [67]. Ren et al. [44] provide a taxonomy of foundation model anomaly detectors as encoders, detectors, or interpreters, highlighting their strengths in anomaly representation, direct detection, or explanation generation. In practice, foundation models enable the detection of anomalies across modalities with minimal supervision. However, pre-trained foundation models may lack domain-specific sensitivity or precision. Recent works demonstrate that specialized prompting and multimodal instruction tuning significantly improve both the detection accuracy and the interpretability of reasoning outputs relative to generic foundation models. For instance, Anomaly-OV [68] integrates GPT-4o with a look-twice feature matching mechanism to achieve strong zero-shot performance and reliable anomaly descriptions on manufacturing benchmarks (e.g., using Anomaly-Instruct-125k and datasets like VisA-D&R), significantly outperforming generic LLMs. Similarly, LogSAD uses a match-of-thought architecture with GPT-4V, CLIP, and SAM to detect both structural and logical defects in industrial images without any training, while also generating compositional rules and calibrated anomaly scores [45].

Beyond CLIP-based and GPT-based foundation models, recent visual large language models (VLLMs) have been specifically adapted for anomaly detection. Myriad [69] integrates a vision expert tokenizer into MiniGPT-4, embedding the segmentation outputs of specialized vision experts into tokens that the LLM can process. AnomalyCLIP [70] introduces object-agnostic prompt learning that captures generic normality and abnormality patterns across categories, achieving strong zero-shot transfer without per-class prompt tuning. VMAD [71] enhances anomaly localization through defect-sensitive structure learning and locality-enhanced token compression, delivering competitive zero-shot performance on MVTec AD and VisA without additional training. More recently, ADSeeker [72] constructed the first visual document knowledge base for industrial anomaly reasoning (SEEK-M&V) and employed a hierarchical sparse prompt mechanism with retrieval-augmented generation, surpassing most AD expert models in zero-shot image-level AUROC. FiLo [73] uses LLM-generated fine-grained anomaly descriptions with position-enhanced localization to achieve state-of-the-art performance.

### 3.2. Cross-Modal Fusion Models

Cross-modal fusion models effectively combine heterogeneous modalities, such as vision, audio, time series, and text during model training or inference to improve the anomaly detection performance. Peng et al. [74] introduce AVadCLIP, which fuses audio and visual inputs via CLIP embeddings, adaptive audiovisual prompts, and uncertainty-driven distillation to enhance detection under noise and occlusion. Ghadiya et al. [61] propose a cross-modal fusion adapter (CFA) with hyperbolic graph attention to dynamically balance audiovisual streams, yielding state-of-the-art weakly supervised video AD performance. Barusco et al. [75] adapt vision-based localization methods to spectrograms, enabling fine-grained temporal-frequency anomaly detection in audio with improved interpretability. Lee et al. [76] present CLIPFUSION, which combines CLIP’s global discriminative features with conditional diffusion-based reconstruction to jointly improve segmentation and classification on the MVTec-AD and VisA datasets. Meanwhile, Wang et al. [77] introduce STADNet, a dual-stream 3D-convolutional model with spatiotemporal attention that fuses RGB and motion for improved surveillance anomaly detection on benchmarks like UCSD Ped2. Finally, Qu et al. [78] propose MFGAN, which integrates temperature, vibration, and acoustic sensor data via attention-based autoencoders to enhance anomaly detection in industrial manufacturing, achieving higher F1 scores than unimodal baselines. These cross-modal fusion methods consistently demonstrate that leveraging complementary modalities significantly enhances anomaly detection performance in both controlled and real-world environments.

### 3.3. Multimodal Augmentation

Multimodal generative frameworks augment anomaly detection pipelines by simulating rare or hard-to-label anomalies using domain knowledge and conditional generation. For instance, key knowledge augmentation (KKA) [79] uses an LLM to synthesize plausible hard vs. easy anomalies based on domain knowledge, enriching the training data and improving classifier boundaries. In audio, FS-TWFR-GMM [80] leverages metadata (e.g., machine types) with diffusion-based generation to simulate anomalies in zero- or few-shot scenarios. Such approaches blur the line between generative modeling and anomaly detection, with LLMs or multimodal models serving both as data generators and reasoning engines. The agentic and multimodal paradigm also extends to more specialized domains. In robotic processes, context-sensitive visual anomaly detection is enabled by combining procedural text with visual inputs through hierarchical VLM prompting [81]. In medical imaging, masked diffusion models trained on healthy scans can detect subtle pathologies without labels [82], while vision language models promise even richer context-aware anomaly detection in future healthcare applications.

### 3.4. Diffusion-Based and Multi-Expert Models

Diffusion models have recently emerged as a powerful paradigm for anomaly detection, leveraging their abilities to learn complex data distributions and produce high-fidelity reconstructions of normal data [22]. A diffusion model trained exclusively on normal samples learns to progressively denoise corrupted inputs; when presented with an anomalous input, the model reconstructs a normal version, and the discrepancy serves as an anomaly score. Several architectures have advanced this paradigm significantly. DiAD [83] introduces a semantic-guided network within a latent diffusion framework for multi-class anomaly detection. MTDiff [84] constructs scale-specific diffusion branches and fuses their outputs to improve pattern coverage across anomalies of varying sizes, outperforming prior methods on diverse benchmarks. CDAD [85] addresses continual anomaly detection by combining a continual diffusion model with an anomaly-masked network, mitigating catastrophic forgetting across sequentially introduced object classes. AnomalyXFusion [86] enhances anomaly synthesis by jointly conditioning on image, text, and mask features within a diffusion framework, enabling more realistic multimodal anomaly generation for data augmentation.

A complementary line of work applies mixture-of-experts (MoE) architectures to anomaly detection, assigning specialized expert networks to handle distinct data distributions or anomaly types. MoEAD [87] incorporates MoE layers into a recursive ViT-based architecture, adaptively selecting feed-forward experts per input token to capture class-specific anomaly semantics while maintaining parameter efficiency. Adapted-MoE [88] uses a routing network to partition same-category samples into subclass-specific feature spaces, with independent expert models constructing separate decision boundaries and a test-time adaptation mechanism to handle distribution shifts. AnomalyMoE [89] dedicates three-level experts to patch, component, and global semantic levels, achieving comprehensive anomaly detection without relying on language guidance. MECAD [90] addresses continual anomaly detection with a multi-expert architecture that adaptively assigns new classes to experts based on feature similarity. These multi-expert approaches are particularly relevant for real-world industrial settings where normal samples exhibit significant intra-class variation and new product types are continuously introduced.

## 4. Multimodal Fusion Methods

**Figure 3:**
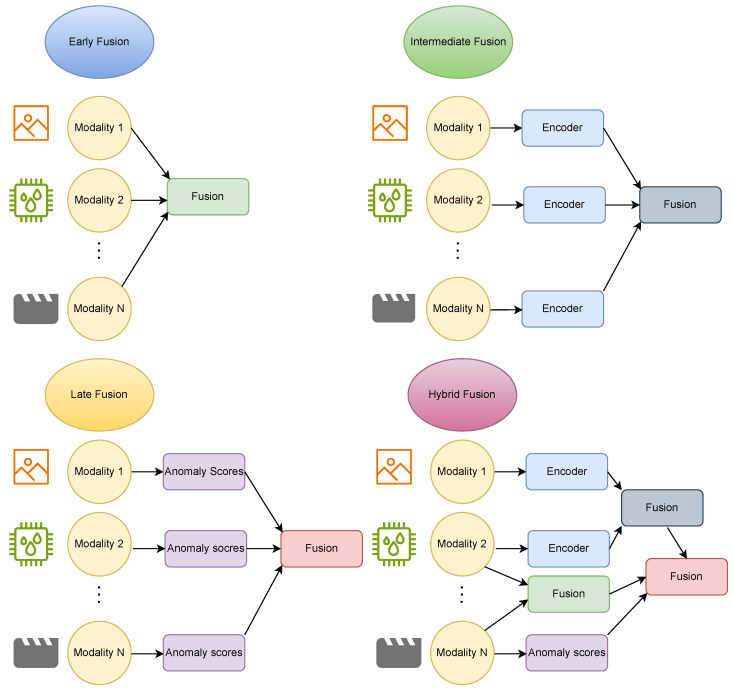
Multimodal fusion methods.

In multimodal anomaly detection, fusion methods determine how data, features, or decisions from each modality are combined to produce an anomaly score or classification. As shown in Figure 3, fusion methods can be categorized based on when the modalities are combined (fusion level) and how they are combined (fusion operation).

### 4.1. Fusion Stages

Fusion stages can be broadly categorized into early (data-level), intermediate (feature-level), and late (decision-level) fusion, depending on the stage at which modalities are combined within the processing pipeline. Recent multimodal anomaly detection systems often combine multiple fusion levels for improved robustness. For example, a system might perform intermediate fusion between closely related modalities while also applying late fusion to incorporate a separate expert model decision. There are also hierarchical fusion architectures that integrate modalities at multiple stages (e.g., fusing some modalities early and fusing again at a higher feature level) to capture both low- and high-level cross-modal interactions. Additionally, multi-task or multi-tier fusion frameworks are emerging, where different fusion stages are optimized for different objectives (for instance, earlier layers fuse modalities for a representation learning task, while later layers fuse decisions for a specific anomaly classification task) [91]. In general, early fusion maximizes direct cross-modal interaction, late fusion maximizes modularity, and intermediate fusion seeks a balance; hybrid approaches leverage the benefits of each. Table 5 summarizes common fusion methods along with their advantages and drawbacks.

#### 4.1.1. Early Fusion (Data Level)

Early fusion concatenates raw or low-level features from all modalities into a joint representation, allowing a single model to learn cross-modal correlations directly. For example, one might concatenate image pixels and depth values channel-wise to form a joint input or merge sensor readings into one feature vector before anomaly modeling. Costanzino et al. [60] propose mapping depth and RGB features via a lightweight cross-modal feature mapper during inference, detecting anomalies through inconsistencies between observed and mapped features. Beyond simple concatenation, some approaches fuse RGB frames with optical flow and depth in a unified tensor before passing through a single network [92]. In log-based monitoring, early fusion has also been applied by integrating multiple system log streams into one high-dimensional feature vector for end-to-end anomaly detection [93]. Early fusion captures direct correlations between modalities from the start but can be insufficient if modalities are not well aligned or if one modality has significantly higher dimensionality (it may dominate the representation) [61]. In practice, early fusion is simple but may force the model to learn a very complex mapping from heterogeneous raw data to anomalies.

#### 4.1.2. Intermediate Fusion (Feature Level)

In feature-level fusion, each modality is first processed by a dedicated encoder to extract higher-level features, and fusion occurs at one or more intermediate layers of the network. For instance, a framework might include a CNN to encode images and an RNN to encode time-series sensor data; their latent feature representations $h^{\left(1\right)}$ and $h^{\left(2\right)}$ are then fused (e.g., concatenated or combined via attention) at a certain layer to produce a joint representation for anomaly detection. This approach allows each modality to contribute more distilled, modality-specific features to the fusion, often making the combined representation more informative [94,95]. The fusion point can be a single layer or multiple layers (multi-stage fusion). A common pattern is a dual-stream (or multi-stream) network that processes modalities separately up to a point and then merges their feature streams. Choosing the appropriate layer (or layers) at which to fuse is critical: fusing too early might mix noisy, incompatible raw signals, whereas fusing too late might fail to capture important cross-modal interactions that could be learned at intermediate levels [64]. Learnable fusion operations such as attention or gating can be inserted at the fusion layer to adaptively weight the contributions of each modality. Intermediate fusion thus aims to balance modality specialization with cross-modal learning.

#### 4.1.3. Late Fusion (Decision Level)

Late fusion involves training separate models for each modality and combining their anomaly scores or decisions via methods such as weighted sums, majority voting, dynamic gating, or mixture-of-experts schemes [96]. This approach treats each modality expert independently up to the decision stage, offering straightforward implementation and modular explainability. It is particularly useful when modalities are largely independent or when safety-critical applications demand that any single modality alone can trigger an alert. However, because it relies on heuristics or separate reconciliation models, late fusion does not learn an integrated cross-modal representation and may struggle when modalities produce conflicting signals. Adaptive late fusion methods, such as mixture-of-experts, address this by learning confidence weights for each expert. For instance, Willibald et al. [29] propose a mixture-of-experts framework combining a Gaussian mixture regression detector on proprioceptive signals with a CLIP-based visual language model, dynamically selecting the most reliable expert at inference. Lin et al. [27] explore the dynamic weighting of video and audio anomaly scores based on environmental conditions.

### 4.2. Fusion Operations/Architectures

Fusion operations or architectures determine how modalities are combined. Formally, let $h^{\left(1\right)}$ and $h^{\left(2\right)}$ be feature representations from two modalities (these could be raw inputs in an early fusion setting or intermediate features in a mid-fusion setting). A fusion operation is a function *F* that combines these representations into a joint feature *z*:

(7) $$z=F(h^{\left(1\right)},h^{\left(2\right)})$$

There are many choices for *F*, ranging from simple arithmetic combinations to complex learned modules. Below, we describe several common fusion operations and modules, along with mathematical formulations and examples from the recent anomaly detection literature.

#### 4.2.1. Concatenation

The simplest fusion operation is to concatenate the two feature vectors (denoted $\left[h^{\left(1\right)};h^{\left(2\right)}\right]$) into one larger vector, which can then be processed by subsequent layers (often, a linear layer or MLP is applied to the concatenated vector to mix the features). For example, one can define

(8) $$z=\phi (\left[h^{\left(1\right)};h^{\left(2\right)}\right]),$$

where $\left[h^{\left(1\right)};h^{\left(2\right)}\right]$ denotes the concatenation of $h^{\left(1\right)}$ and $h^{\left(2\right)}$, and ϕ could be an identity function (direct concatenation) or a learnable transformation (a fully connected layer). Several multimodal networks use simple concatenation followed by feed-forward layers at the fusion stage [64]. Concatenation preserves all information from both modalities, but it assumes that the features are already aligned or comparable in scale, and the burden is on the subsequent layers to identify cross-modal relationships.

#### 4.2.2. Element-Wise Addition or Weighted Sum

Another common fusion method is the element-wise addition of the feature vectors. If $h^{\left(1\right)}$ and $h^{\left(2\right)}$ are of the same dimensionality, one can fuse them by summing corresponding elements: $z=h^{\left(1\right)}+h^{\left(2\right)}$. More generally, a weighted sum allows the model to learn the contribution of each modality:

(9) $$z=w_{1}⊙h^{\left(1\right)}+w_{2}⊙h^{\left(2\right)},$$

where $w_{1}$ and $w_{2}$ are scalar weights or weight vectors (learned or set by prior knowledge), and ⊙ denotes element-wise multiplication (scaling each feature). This operation effectively averages or emphasizes modalities in each feature dimension. Addition-based fusion is computationally cheap and treats the two feature sets symmetrically. For instance, some network architectures treat a secondary modality as an extra channel appended to the primary modality and use element-wise addition in intermediate layers to blend the information [60]. A variant of weighted sum fusion is to use a gating mechanism that dynamically adjusts weights based on the data. In a gating approach, a function (often a small neural network with a sigmoid activation) computes a gating factor α between 0 and 1 from the inputs:

(10) $$\alpha =\sigma \left(W\;[\;h^{\left(1\right)};\;h^{\left(2\right)}\;]\right),$$

where *W* is a learnable weight matrix and σ is the sigmoid function. Then, the fused output is

(11) $$z=\alpha ⊙h^{\left(1\right)}+\left(1-\alpha \right)⊙h^{\left(2\right)}.$$

Here, α (which could be a single scalar or a vector of gating values for different feature dimensions) acts as an adaptive trade-off: if α is close to 1, modality 1 dominates, and, if α is close to 0, modality 2 dominates. This type of gating is used in some attention fusion modules; for example, the cross-modal feature attention (CFA) module in an audiovisual fusion model learns a dynamic weight to balance audio and video features for each time frame [61].

#### 4.2.3. Multiplicative or Bilinear Fusion

Multiplicative fusion involves combining features by multiplication interactions rather than addition. The simplest form is element-wise multiplication:

(12) $$z=h^{\left(1\right)}⊙h^{\left(2\right)},$$

which yields a fused feature where each dimension $z_{i}=h_{i}^{\left(1\right)}\cdot h_{i}^{\left(2\right)}$. This operation can highlight feature dimensions that strongly agree across modalities (if both $h_{i}^{\left(1\right)}$ and $h_{i}^{\left(2\right)}$ are high) or dampen those that disagree in sign. Element-wise multiplication requires the two feature vectors to be the same size and implicitly assumes a one-to-one correspondence between their dimensions. A more general form is bilinear fusion, where each feature in one modality is multiplied by each feature in the other modality (an outer product), potentially with learnable weights. Bilinear fusion is defined as

(13) $$z_{jk}=h_{j}^{\left(1\right)}\cdot h_{k}^{\left(2\right)},$$

which produces a matrix capturing all pairwise interactions between elements of $h^{\left(1\right)}$ and $h^{\left(2\right)}$. This outer product can then be vectorized or passed through pooling layers to form the final fused representation. In practice, it is common to introduce a parameterized bilinear form to control the dimensionality, such as

(14) $$z={\left(h^{\left(1\right)}\right)}^{T}\;W\;h^{\left(2\right)},$$

where *W* is a learned matrix or tensor. This yields each component of *z* as a weighted combination of pairwise products of $h^{\left(1\right)}$ and $h^{\left(2\right)}$ elements. Bilinear fusion can capture complex interactions between modalities and has been employed in some vision language models and multimodal classifiers [97,98]. However, it typically produces very high-dimensional representations (especially if we explicitly take an outer product) and can easily overfit without large amounts of data or proper regularization.

#### 4.2.4. Attention-Based Fusion

Attention mechanisms adaptively fuse modalities by weighting relevant features from one modality using another. In a cross-attention setup, one modality’s features (the query set) are used to softly select or weight the features of another modality (the key and value set). Formally, given two sets of features $H^{\left(1\right)}=\left\{h_{i}^{\left(1\right)}\right\}$ and $H^{\left(2\right)}=\left\{h_{j}^{\left(2\right)}\right\}$, attention-based fusion can compute, for each feature $h_{i}^{\left(1\right)}$, a weighted combination of the $H^{\left(2\right)}$ features,

(15) $$\mathrm{Attn}\left(h_{i}^{\left(1\right)},H^{\left(2\right)}\right)=\sum_{j}\alpha_{ij}\;h_{j}^{\left(2\right)},$$

where the attention weights $\alpha_{ij}$ are given by

(16) $$\alpha_{ij}={\mathrm{softmax}}_{j}\;\left(h_{i}^{\left(1\right)}W_{Q}\;{\left(h_{j}^{\left(2\right)}W_{K}\right)}^{T}\right)$$

in a typical formulation (with $W_{Q},W_{K}$ projection matrices for queries and keys, respectively). The result is that each element of one modality can attend to (i.e., extract information from) the elements of another modality that have high content similarity or relevance. The fused representation could be, for instance, the original $H^{\left(1\right)}$ features augmented with these attention outputs, or vice versa. To reduce the computational cost, attention bottlenecks introduce *B* learnable latent vectors $b_{1},\ldots ,b_{B}$ as intermediaries [94]. Each modality attends to these bottlenecks (at most N×B interactions) and then reverse-attends back, enforcing a compact shared representation. This two-step bottleneck attention generalizes frameworks such as LB3M for RGB + 3D fusion in industrial anomaly detection [60], yielding efficient and effective cross-modal integration. Cross-attention is ideal when modalities align semantically (e.g., synchronized video–audio or image–text). For time-series anomaly detection, MST-GAT uses intra- and inter-modal graph attention to learn sensor correlations and improve anomaly scoring [57]. In video surveillance, stacked cross-attention layers fuse RGB, optical flow, and audio, dynamically weighting cues for superior detection [92,99].

#### 4.2.5. Feature Mapping

Rather than fusing features through arithmetic or attention, this approach uses one modality to reconstruct or predict the features of another. A mapping function *M* is trained to translate from one modality to another, $M:h^{\left(1\right)}\mapsto {\hat{h}}^{\left(2\right)}$, where ${\hat{h}}^{\left(2\right)}$ is the predicted counterpart of $h^{\left(2\right)}$. During inference, the reconstruction error $|{\hat{h}}^{\left(2\right)}-h^{\left(2\right)}|$ serves as an anomaly score, i.e., large deviations indicate cross-modal inconsistency and potential anomalies. This method exploits the idea that, under normal conditions, modalities agree and can predict each other well. For example, Costanzino et al. map depth to RGB features and vice versa, detecting anomalies when predictions and observations diverge [60]. Beyond direct mapping, more advanced methods integrate contrastive learning and memory mechanisms. Wang et al. propose M3DM for RGB + 3D anomaly detection using patch-wise contrastive learning to align 3D point cloud and image features in a shared embedding space [63]. In general, these methods detect novelties by evaluating how well one modality can explain the other. A failure to do so suggests a modality-specific anomaly that would be missed in unimodal analysis.

#### 4.2.6. Modality and Temporal Alignment

Effective fusion often requires aligning modalities spatially, semantically, and temporally before combining them. In spatial or feature-wise alignment, inputs $x_{1},x_{2}$ are transformed via $f_{1}\left(x_{1}\right)$ and $f_{2}\left(x_{2}\right)$ into a common space (e.g., projecting depth maps or point clouds onto the image plane so that each pixel’s depth and RGB correspond) [60]. Alternatively, contrastive or correlational objectives (e.g., contrastive loss) train feature extractors so that paired inputs yield embeddings $h^{\left(1\right)}$ and $h^{\left(2\right)}$ that coincide, while mismatches diverge. Temporally, modalities sampled at different rates or with offsets must be synchronized. A basic strategy resamples or buffers slower streams (e.g., holding the latest $h^{\left(2\right)}$ constant for multiple $h^{\left(1\right)}$ frames), creating aligned pairs $\left(h_{t}^{\left(1\right)},h_{\left\lfloor t/r\right\rfloor }^{\left(2\right)}\right)$. More advanced methods employ temporal attention or sequence alignment when events lag across sensors, letting each feature at a time *t* attend over a window of the other modality timestamps.

#### 4.2.7. Graph-Based Fusion

Graph-based fusion leverages graph neural networks (GNNs) to integrate multimodal information by modeling modalities or components as nodes in a graph, with edges capturing relationships—whether physical, functional, or learned. A GNN then propagates and aggregates information across this graph to produce fused node- or graph-level embeddings. In multimodal anomaly detection, each modality (sensor) can be treated as a node, with edges encoding inter-modality dependencies. Through message passing, each node updates its state by blending its own features with those of its neighbors:

(17) $$h_{u}^{\left(l+1\right)}=\sigma \;\left(W\;h_{u}^{\left(l\right)}+\sum_{v\in N(u)}\Theta \;h_{v}^{\left(l\right)}\right),$$

where $h_{u}^{\left(l\right)}$ is the representation at layer *l*; N(u) are neighbors; W,Θ are learnable weights; and σ is an activation function. Ektefaie et al. apply this approach to sensor graphs, learning node embeddings that capture both individual and contextual information for anomaly detection [100]. Passos et al. extend this with CCA-GNN, which jointly optimizes GNN embeddings, and canonical correlation analysis to enforce cross-modal alignment, helping to detect anomalies that disrupt typical inter-modality correlations [101]. Xia et al. propose VLDFNet, which builds a view graph over 2D projections of 3D data and fuses image and point cloud features via a disentangled latent space [102]. The graph structure enables the modeling of both intra- and inter-modal relationships, enhancing robustness in anomaly detection. In general, graph-based fusion is well suited for scenarios where relations between multiple modalities are key to understanding anomalies. It flexibly encodes multimodal interactions by treating modality as another dimension of connectivity in the graph.

### 4.3. Fusion Design Principles and Trade-Offs

Designing effective multimodal fusion requires balancing integration and modularity: modalities should inform each other without allowing noise or irrelevant signals to degrade performance. Recent research suggests several key design principles.

- Fuse at Multiple Levels: Combining mid-level feature fusion with late-stage score fusion enhances robustness. Early fusion may overlook modality-specific noise, while multi-scale fusion captures both structural and semantic information [64].
- Selective Feature Fusion: Not all layers contribute equally to cross-modal alignment. Shallow or mid-level features often provide better spatial or temporal grounding across modalities than very early or late representations.
- Learnable and Adaptive Fusion: Models that use attention, gating, or mixture-of-experts mechanisms dynamically adjust modality contributions, outperforming static fusion strategies. This adaptiveness is especially valuable in heterogeneous or noisy environments [61].
- Efficiency: Fusing mid-level or compact embeddings is computationally efficient compared to raw input fusion. Techniques such as bottleneck layers and dimensionality reduction maintain informativeness while reducing overhead.
- Domain-Specific Design: Fusion should be tailored to the task and data characteristics. For instance, if one modality is unreliable or frequently missing, late fusion provides robustness. If anomalies depend on fine-grained correlations across streams (e.g., visual flashes coinciding with audio spikes), then mid-level feature fusion is essential. Domain knowledge can guide initial architecture choices, with further refinement via ablation or architecture search.

In general, multimodal fusion is not a one-size-fits-all problem. The optimal strategy depends on the nature of the modalities, the anomaly types, and the computational constraints of the deployment scenario.

## 5. Multimodal Datasets and Benchmarks

Multimodal anomaly detection relies on rich datasets that align multiple sensor modalities such as vision, audio, LiDAR, and text, yet truly multimodal benchmarks remain relatively scarce. Recent progress, however, has led to important datasets featuring synchronized multimodal recordings with detailed ground truth annotations. These datasets typically combine modalities like RGB and LiDAR for driving scenarios, audio and video streams, RGB images with depth information, and multi-sensor industrial data. Existing benchmarks often lack standardized evaluation metrics and underrepresent certain modality combinations, such as vision–text fusion in industrial anomaly detection or sensor–metadata fusion in manufacturing. The MMAD dataset [103] marks progress toward multimodal language model evaluations, although it primarily emphasizes vision. Table 6 presents multimodal datasets and benchmarks, with details including the modality, size, anomaly types, and label information.

## 6. Challenges, Solutions, and Future Works

Although agentic and multimodal anomaly detection systems provide robust anomaly detection, their deployment faces challenges, including heterogeneous data fusion, data scarcity, and high inference costs [110]. LLM-based agentic pipelines compound these issues with added complexity surrounding agent coordination, trust, and security [41]. This section outlines key challenges, solutions, and future research directions. Table 7, presents a summary of main challenges in multimodal/agentic anomaly detection and prospective research directions.

### 6.1. Data Scarcity

Anomalies are inherently rare, leading to severe data imbalances and the scarcity of labeled examples for training. This scarcity impedes the learning of robust detectors and often biases models toward the abundant normal patterns. A promising solution to such a problem is synthetic anomaly generation using generative models. Early approaches employed GANs to perturb or reconstruct normal data, thereby creating pseudo-anomalies that enrich the training distribution [111]. More recently, diffusion models have shown the ability to produce high-fidelity synthetic anomalies [112,113].

In the multimodal setting, AnomalyXFusion [86] demonstrates that jointly conditioning on image, text, and mask features within a diffusion framework produces more realistic and diverse synthetic anomalies, while multi-expert diffusion architectures such as MTDiff [84] improve pattern coverage by operating at multiple scales. Despite progress, ensuring that synthetic anomalies truly reflect real-world outliers remains a challenge. Future research should refine generative processes using more advanced conditional generation (e.g., text- or class-conditioned prompts to specify the anomaly type) and hybrid GAN–diffusion pipelines.

### 6.2. Multimodal Representation and Alignment

Multimodal anomaly detection requires integrating multiple data modalities into a unified representation, typically leveraging contrastive learning and attention mechanisms to align heterogeneous features [114]. Despite recent advances, modality imbalance and naive feature fusion remain major challenges, often suppressing modality-specific anomalies or limiting subtle cross-modal interactions [114]. Methods such as AVadCLIP and adaptive cross-modal fusion adapters dynamically balance and align modalities by adaptively weighting features based on context [61,74]. Future research must further develop adaptive fusion methods and robust alignment techniques capable of effectively handling missing or noisy modalities.

### 6.3. Agentic Reasoning and LLM Limitations

LLM-based agents enhance interpretability and reasoning but are prone to hallucination and domain mismatches [115]. Misinterpretations in specialized domains highlight the need for domain-adapted LLMs and structured knowledge integration. Future work should enhance LLM factuality and reasoning alignment and develop benchmarks for the evaluation of agent reasoning fidelity.

### 6.4. Real-Time Inference and Scalability

For several practical applications (e.g., security monitoring, autonomous driving), anomaly detection must operate under strict latency and throughput constraints. Complex agent frameworks can be computationally expensive, limiting their real-time use. Recent work has highlighted that detectors often involve very large networks, which are not suitable for cost-effective real-time applications [116]. A promising future direction is model compression and efficiency enhancement. Techniques such as knowledge distillation have demonstrated the ability to transfer anomaly detection capabilities from a heavy teacher model to a lightweight student, achieving similar accuracy with dramatically fewer parameters. Future work should explore dynamic model cascades, online model adaptation for data streams, and hardware-friendly implementations (e.g., via model quantization or edge computing) to ensure scalability.

### 6.5. Interpretability

Interpretability remains a major challenge in anomaly detection, particularly in high-stakes scenarios. Current approaches focus on generating natural-language rationalizations or employing explainable rule-based reasoning to enhance transparency [115]. Additionally, agent traceability, where each decision or action is logged and auditable, is increasingly emphasized to validate anomaly detection processes. Future work should explore the development of intuitive interfaces for anomaly explanation, the effective communication of uncertainty, and methods ensuring the reliability and trustworthiness of explanations provided to end-users [115].

### 6.6. Evaluation and Benchmarking

Evaluation and benchmarking in multimodal and agentic anomaly detection face significant limitations due to the lack of standardized benchmarks and consistent metrics, resulting in difficulties in comparing methods across studies [115]. Recent efforts such as the MMAD [103] and AnoVox [104] datasets partially address these gaps but remain domain-specific. To progress, the field requires standardized anomaly taxonomies, evaluation protocols, and benchmarks that can handle complex multimodal and dynamic scenarios.

### 6.7. Theoretical Foundations

The theoretical underpinnings of agentic and multimodal anomaly detection remain insufficient, often relying on empirical heuristics. While progress has been made using graph-based anomaly detection for modeling agent interactions [41], there is a pressing need for rigorous theoretical frameworks that can characterize detector behavior under diverse conditions, establish formal performance bounds, and analyze the complex dynamics of multi-agent systems.

## 7. Conclusions

Anomaly detection is evolving rapidly to meet the demands of increasingly complex, heterogeneous, and dynamic real-world environments. As intelligent systems become more autonomous and multimodal by design, the integration of agentic reasoning with cross-modal perception is not only natural but necessary. This work has presented a comprehensive overview of recent advances at the intersection of multimodal and agentic anomaly detection—two emerging paradigms that enable more robust, interpretable, and context-aware detection systems. We review foundational work in anomaly detection and highlight the shift from unimodal, static methods to multimodal fusion frameworks and reasoning-capable agents. We introduce a taxonomy that classifies agentic anomaly detection systems by architecture, capability, and modality scope, and we examine recent benchmarks, tools, and datasets that are driving this research frontier. We also identify core challenges and discuss current mitigation strategies and promising research directions. Future research should prioritize adaptive fusion strategies, theory-grounded agent design, and secure, explainable deployments. We hope that this work serves as a foundation for researchers and practitioners seeking to develop next-generation anomaly detection systems that are both perceptually rich and autonomously intelligent.

## Figures and Tables

**Table 1:** Taxonomy of agentic anomaly detection methods, grouped by agent architecture, key capabilities, data modality, and evaluation datasets.

Framework	Agent Type	Capabilities	Modality/Scope	Evaluation/Dataset
ARGOS [39]	Planner (multi-agent LLM)	Workflow planning, tool use, external retrieval	Multimodal (TS + logs + web-text + meta)	KPI, Yahoo, internal Microsoft data
AD-Agent [40]	Multi-agent (LLM pipeline)	Instruction parsing, model selection, code generation	Tabular/graph/time series	ADBench
SentinelAgent [41]	Oversight (LLM tool user)	Graph modeling, oversight, cognitive inconsistency detection	Multimodal logs + plans + MAS interactions	Simulated email assistant; Magnetic-One
Audit-LLM [42]	Planner (multi-agent)	Task decomposition, feedback, log auditing	Logs + metadata	Cybersecurity benchmarks
IBM Shipping [43]	Single-agent (LLM + tools)	Reasoning on multimodal sensor/knowledge graph (KG) data	Sensor + KG (maritime)	Real shipping operation logs
AnomalyRuler [44]	Single-agent (reasoning)	Rule induction, chain-of-thought reasoning	Video	Few-shot video AD benchmarks
LogSAD [45]	Single-agent (reasoning)	Compositional vision–language reasoning (GPT-4V + CLIP)	Mixed (image + text)	Industrial image and text AD tasks
VLM4TS [46]	Tool-using agent	Vision–language transformation, retrieval-augmented AD	Multimodal (time series + text)	Time-series AD benchmarks
AESOP [47]	Single-agent (LLM)	Fast anomaly classification with fallback planning	Visual (robotics)	Quadrotor and vehicle simulations
ALFA [48]	VLM (LLM + vision)	Zero-shot visual anomaly detection via prompts	Visual (images)	MVTec, VisA anomaly datasets
MAS-LSTM [49]	Multi-agent (LSTM)	Local LSTM voting-based fusion	Time series (IIoT)	Industrial IoT traffic
MARL [50]	Multi-agent (RL)	Decentralized RNN predictors, normality scoring	Observations (MARL)	StarCraft (multi-agent env.)
RL-IDS [51]	Multi-agent (RL)	Parallel DQN agents, cost-sensitive learning	Network traffic	CIC-IDS-2017 network dataset
GPT-4 Prompt [52]	Detection-only agent	Zero-shot anomaly classification via prompting	Time series, text	Prompt-based scoring benchmarks

**Table 2:** Agentic anomaly detection paradigms by agent capability, with representative examples.

Agent Type	Key Capability	Examples	Strengths	Limitations
Detection-Only Agents	Direct labeling via prompt or model output	SigLLM [53]; GPT-4V zero-shot [52]	Simple deployment, fast inference	Prone to LLM errors (hallucinations), no deep reasoning
Reasoning Agents	Chain-of-thought, rule induction from few-shot normals	AnomalyRuler [44]; LogSAD [45]	Explainable decisions; uses in-context learning	Sensitive to prompt design; needs clean normal data
Tool-Using Agents	External API or tool integration	ARGOS [39]; VLM4TS [46]; SentinelAgent [41]	Context-aware and domain-grounded	Tool dependency; higher latency and complexity
Planner Agents	Workflow decomposition, memory usage, multi-step planning	Audit-LLM [42]; ARGOS pipeline; LLM-based FM [3]	Tackles complex multi-stage tasks; dynamic adaptation	Complex architecture; more costly to develop/maintain

**Table 3:** Systematic comparison of reasoning agents and planner agents in agentic anomaly detection.

Dimension	Reasoning Agent	Planner Agent
Core mechanism	Single-pass chain-of-thought or rule induction: $c_{1:T}=\pi \left(x\right),\;y=f\left(x,c_{1:T}\right)$	Multi-step workflow optimization: $\Pi^{*}=\arg\max_{\Pi \in S}U\left(\Pi ;x\right)$ with action sequence $\Pi =[a_{1},\ldots ,a_{n}]$
Control flow	Linear (forward-only); no branching or looping	Iterative with branching, looping, and conditional replanning based on intermediate results
Tool/subagent use	None or minimal; reasoning is self-contained within the LLM context	Central capability; orchestrates external tools, databases, subagents, and APIs
Adaptivity	Static once the reasoning chain is generated; no revision of earlier steps	Dynamic; can revise the plan, requery tools, or trigger deeper analysis based on intermediate outcomes
State management	Stateless beyond the current reasoning trace	Maintains explicit state (memory, task queues, partial results) across steps
Typical output	Anomaly label + human-readable reasoning trace (explanation)	Anomaly decision + structured investigation report with evidence from multiple sources
Latency	Low (single LLM forward pass or few-shot inference)	Higher (multiple sequential or parallel steps, tool calls, possible replanning)
Complexity	Moderate; prompt design is the main engineering effort	High; requires workflow specification, error handling, agent coordination
Representative examples	AnomalyRuler [44]: induces rules from normal video and then applies them in one pass; LogSAD [45]: compositional vision–language reasoning without training	Audit-LLM [42]: coordinator decomposes queries → executor runs them → critic validates → loop until satisfied; ARGOS [39]: iteratively generates, validates, and refines detection rules across agents
When to use	Anomalies can be identified by logical inspection of the input; explainability is prioritized over exhaustive investigation	Complex, multi-source anomalies requiring evidence gathering, hypothesis testing, or iterative refinement

**Table 4:** Multimodal anomaly detection methods and their innovations.

Method	Modalities	Key Idea	Benchmark	Quantitative Results
BTF [62]	RGB + 3D (depth)	First RGB-3D industrial AD; combines 3D handcrafted features (FPFH) with pre-trained 2D CNN; memory bank of normal feature patches.	MVTec 3D-AD	I-AUROC 87.3%, P-AUROC 99.3%, AUPRO 96.4%
M3DM [63]	RGB + 3D	Frozen ViT + Point-MAE; hybrid fusion with contrastive-learned fusion memory bank; point-level alignment.	MVTec 3D-AD	I-AUROC 94.5%, P-AUROC 99.3%, AUPRO 96.1%
CFM [60]	RGB + 3D	Learns cross-modal feature mapping (2D↔3D) on normal data; memory bank-free; anomalies detected via mapping disagreement.	MVTec 3D-AD	I-AUROC 95.4%, P-AUROC 99.3%, AUPRO 97.0%
CPIR [59]	RGB + 3D	Bidirectional feature mapping with autoencoder reconstruction and shared latent bridge (LB3M) for cross-modal consistency.	MVTec 3D-AD	I-AUROC surpasses CFM; SOTA on detection and segmentation (full- and few-shot)
3D-ADNAS [64]	RGB + 3D	Neural architecture search for optimal multimodal fusion; two-level search (intra- and inter-module).	MVTec 3D-AD	I-AUROC 95.1% (+0.6% over M3DM with 25× less memory)
WS-VAD [61]	Video + Audio	Cross-modal fusion adapter (CFA) gates noisy modalities; hyperbolic graph attention (HLGAtt) links segments.	XD-Violence	AP 86.34% (+0.67% over prior SOTA) on XD-Violence for violence detection
AnomalyGPT [65]	Image + Text	LVLM-based AD (MiniGPT-4); prompted fine-tuning maps simulated anomalies to text; image decoder for localization.	MVTec AD, VisA	1-shot: Acc 86.1%, I-AUC 94.1%, P-AUC 95.3% (MVTec AD)
LAD-Reasoner [66]	Image + Text	Tiny (3B) multimodal LM for logical AD with natural language explanations; SFT + GRPO reinforcement.	MVTec LOCO AD	Matches Qwen2.5-VL-72B in accuracy/F1; outperforms AnomalyGPT and APRIL-GAN on all 5 LOCO categories

**Table 5:** Fusion methods in multimodal anomaly detection.

Fusion Method	Description	Pros	Cons
Early Fusion	Merge raw inputs or low-level features; then, a single model processes them, e.g., treat LiDAR depth as extra image channels.	Captures raw cross-modal correlations; simple implementation.	Modalities must be aligned; model may be overwhelmed by heterogeneous input.
Intermediate Fusion	Separate encoders, fuse at intermediate layer(s) via concat, add, attention, etc.	Balances modality specialization and interaction; learnable fusion can emphasize important features.	Need to choose when and how to fuse (hyperparameters); improper fusion point can harm performance.
Late Fusion	Independent anomaly scores or decisions per modality, combined at end (e.g., weighted average or voting).	Each modality can be optimized/tuned separately; interpretable contributions; robust if one modality fails (others still contribute).	Loses benefit of joint feature learning; needs method to set weights or logic for combining decisions.
Hybrid/Multi-Stage	Fuse at multiple points or use a mix of the above (including multimodal transformers).	Very flexible, can capture both low-level and high-level interactions; often highest accuracy.	Increased complexity; requires sufficient data; harder to interpret and configure.

**Table 6:** Multimodal anomaly detection datasets.

Dataset	Modalities	Size/Scale	Anomaly Types	Ground Truth
AnoVox [104]	RGB + LiDAR	City-scale driving (multi-sensor)	Spatial and temporal road anomalies	Voxel-level segmentation
MAVAD [105]	Video + Audio	764 videos (11 classes)	Traffic anomalies (e.g., U-turns, obstructions)	Clip-level labels
MVTec LOCO [106]	RGB images	3644 images (5 categories)	Structural and logical anomalies	Pixel-level masks
MVTec 3D-AD [107]	RGB + depth (3D)	4000+ high-resolution scans (10 categories)	Surface and depth irregularities	2D masks + precise depth
MMAD [103]	RGB + text prompts	8366 images with 39,672 QA pairs	Caption-based AD behaviors	QA accuracy and response correctness
AURSAD [108]	Multi-sensor time series	2045 samples	Robot screw driving anomalies	Sample-level labels
DoTA [109]	Video	4677 dashcam clips	Traffic accidents/anomalies	Temporal, spatial, categorical

**Table 7:** Key challenges in multimodal/agentic anomaly detection and prospective research directions.

Challenge	Current Solution	Future Directions
Data scarcity	Synthetic anomaly generation via GANs/diffusion, data augmentation	Advanced conditional generation (text/prompts), hybrid GAN–diffusion approaches, few-shot simulation
Modality alignment	Cross-modal embeddings (CLIP, contrastive losses), feature fusion layers	Unified multimodal representations (transformers), dynamic fusion strategies, multimodal foundation models
LLM domain mismatch	Prompt engineering, few-shot normal exemplars, rule-based prompting	Domain-adapted LLMs, hybrid neuro-symbolic systems, anomaly-aware fine-tuning
Real-time operation and scalability	Knowledge distillation (LLM to lightweight student), fast/slow pipelines	Model compression, efficient LLM architectures, adaptive inference scheduling
Benchmarking and evaluation	Emerging datasets (MMAD, AnoVox)	Comprehensive multimodal AD benchmarks and metrics, standardized anomaly taxonomies
Theoretical foundations	Ad hoc frameworks (e.g., graph models for multi-agent systems)	Formal analysis of LLM–agent behavior, robustness theory, multi-agent anomaly theory

## Data Availability

The data presented in this study are available at the sources mentioned in the text.
